# Etat nutritionnel des enfants âgés de 6 à 59 mois infectés par le VIH mais non traités aux ARV à Lubumbashi

**DOI:** 10.11604/pamj.2014.19.7.3932

**Published:** 2014-09-04

**Authors:** Costa Kazadi Mwadianvita, Faustin Ngoy Kanyenze, Cecile Watu Wembonyama, Florence Mujing A Mutomb, Kalombo Mupoya, Albert Mwembo–Tambwe A Nkoy, Prosper Kalenga Mwenze

**Affiliations:** 1 Université de Lubumbashi, Faculté de Médecine, Département de Sciences Biomédicales, Lubumbashi, République Démocratique du Congo; 2 Université de Lubumbashi, Faculté de Médecine, Département de Pédiatrie, Lubumbashi, République Démocratique du Congo; 3 Université de Lubumbashi, Faculté de Médecine, Ecole de Santé Publique, Lubumbashi, République Démocratique du Congo; 4 Université de Lubumbashi, Faculté de Médecine, Département de Gynécologie Obstétrique, Lubumbashi, République Démocratique du Congo

**Keywords:** Etat nutritionnel, enfant, infection par le VIH, Lubumbashi, nutritional status, child, HIV infection, Lubumbashi

## Abstract

**Introduction:**

L'infection par le VIH provoque et/ou aggrave les déficits nutritionnels de l'enfant. Ce travail avait pour objectif d'analyser l’état nutritionnel des enfants infectés par le VIH à Lubumbashi.

**Méthodes:**

Une étude transversale portant sur 83 enfants âgés de 6 à 60 mois s'est déroulée de mai 2010 à mai 2011 dans trois(3) centres de prise en charge des Personnes Vivant avec le VIH(PVV), notamment le Centre d'Excellence(CE) de l'hôpital Sendwe, le Centre Amo-Congo de la Kenya et le Centre de Référence de la Kenya. Les statistiques descriptives usuelles ont été utilisées.

**Résultats:**

La prévalence de la malnutrition globale était de 60,2% (n = 50) dont 8,4% de malnutrition sévère. Le poids moyen était de 11,6±4,1 kg avec un minimum de 5 kg et un maximum de 22 kg. Le taux d'hémoglobine moyen était d'environ 9,8± 2,0 g/dl avec une prévalence globale de l'anémie (hémoglobine < 11g/dl) à 69,9%. L’émaciation concernait 20,5% des enfants et 8,4% avaient un retard de croissance. Le retard de croissance (p = 0,007), l'insuffisance pondérale (p = 0,002) et l’émaciation (p = 0,046) étaient associés de façon significative à l’état avancé de l'infection à VIH. La survenue de l'anémie n’était pas associée au déficit nutritionnel (p = 0,6).

**Conclusion:**

Ces résultats révèlent que l'infection à VIH modifie l’état nutritionnel des enfants à Lubumbashi avec 60,2% de malnutrition globale et 8,4% de retard de croissance. Les enfants au stade avancé de l'infection à VIH en sont plus affectés.

## Introduction

La malnutrition et le VIH/SIDA sont intimement liés et, ensemble, ils représentent un défi important pour la santé communautaire. La malnutrition est un marqueur de mauvais pronostic chez les enfants infectés par le VIH [1]. En Afrique sub-saharienne, la prévalence de la malnutrition est influencée par une multitude de facteurs dont l'infection à VIH, les maladies infectieuses chroniques. En 2005, 25% de la population mondiale était malnutrie [2]. Cela représente une augmentation de 43 millions de personnes depuis 1990. L′Afrique australe a la plus forte prévalence de la malnutrition (37%), suivie par l′Afrique de l'Est (35%), l′Afrique centrale 30%, et l'Afrique de l′Ouest (14%) [2]. En République Démocratique du Congo (RDC), les diverses études réalisées sur la prévalence de Malnutrition Protéino-Energétique (MPE) chez les enfants de 0 à 5 ans ont montré que 34% des enfants souffrent d′une insuffisance pondérale, 45% de retard de croissance et de 10% d′émaciation [3]. L'infection par le VIH est souvent associée à des carences nutritionnelles chez les enfants [4] et de sa part, la dénutrition aggrave la maladie, accroit la morbidité et diminue la survie [5]. Etant multifactorielle, à travers l'apport alimentaire insuffisant, les infections opportunistes, les parasitoses intestinales, l'inefficacité du métabolisme due à l'activité des cytokines et des diarrhées, la malnutrition induit l'immunodépression et aggrave celle induite par l'infection à VIH [6]. L'objet de cette étude était d'analyser l’état nutritionnel des enfants âgés de moins de 5 ans infectés par le VIH mais naïfs au traitement antirétroviral à Lubumbashi.

## Méthodes

**Cadre de l’étude** Cette étude a été menée à Lubumbashi, en RD Congo, dans trois(3) centres de prise en charge des Personnes Vivant avec le VIH(PVV), notamment le Centre d'Excellence de l'hôpital Sendwe, le Centre Amo-Congo de la Kenya et le Centre de Référence de la Kenya. Ces trois formations médicales assurent les consultations et la prise en charge des PVV sans exclusivité d’âge et de sexe. Contrairement aux formations médicales d'Amo-Congo et de Kenya où la majorité des malades qui consultent est de niveau social bas, le centre d'excellence de l'hôpital Sendwe accueille une classe sociale relativement aisée.

### Type et période de l’étude

Il s′agit d′une étude transversale descriptive qui s'est déroulée de Mai 2010 à Mai 2011 soit une période de 12 mois.

**Population d’étude:** Elle était constituée de 83 enfants âgés de 6 à 59 mois et pris en charge aux trois centres précités. Etaient inclus dans l’étude, les enfants séropositifs au VIH1 ou VIH2, naïfs au traitement antirétroviral, étant suivis régulièrement.

**Variables:** 1) Sociodémographiques: les paramètres utilisés étaient l’âge et le sexe; 2) Cliniques et biologiques: les enfants infectés par le VIH ont été classés selon le stade clinique de l'infection en fonction des critères de l'OMS 2006 [7]. D'autres variables ont été analysées dont les circonstances de découverte du VIH, le mode de contamination. Dans les laboratoires bien équipés, la progression du VIH est essentiellement contrôlée par le nombre de CD4 (chez les enfants, en pourcentage des CD4) alors que dans les pays à ressources limitées comme le nôtre, le dosage des CD4 n'est pas accessible pour tous les enfants. Ainsi les décisions de gestion ont été guidées par le stade clinique. La prise en charge en ambulatoire comprenait la surveillance clinique et biologique, le traitement des infections mineures, la prophylaxie au cotrimoxazole. La prise en charge nutritionnelle est quasi inexistante. Pour la classification de l'anémie, elle a été définie selon les critères de l'OMS par un taux d'hémoglobine inférieur à 11 g/dl. Elle est considérée comme sévère à un taux d'hémoglobine inférieur à 7,0 g/dl. Elle est modérée si ce taux se situe entre 7,0 g/dl et 9,9 g/dl. Et l'anémie est considérée comme légère si ce taux se situe entre 10 g/dl et 11g/dl [8].

**Anthropométriques:** Le poids des enfants dévêtus a été mesuré à l'aide d'une balance Salter Germany (Model 750) étalonnée régulièrement. L'aiguille de la balance était remise à zéro avant la pesée. La taille des enfants a été déterminée, par deux enquêteurs, en utilisant un mètre ruban en position horizontale. Les classifications [9] de Waterlow et de Gomez ont été utilisées pour évaluer l’état nutritionnel des enfants. L’âge, le sexe, la taille et le poids prélevés ont permis de calculer l'indice poids pour âge (PPA) pour rechercher l'insuffisance pondérale ou la malnutrition globale, l'indice taille pour âge (TPA) pour rechercher le retard de croissance ou la malnutrition chronique et l'indice poids pour taille (PPT) pour rechercher l’émaciation ou la malnutrition aiguë. Ces indices étaient exprimés en « Z-score ». Celui-ci représente un écart de la mesure de l'enfant par rapport à la médiane de référence divisé par l’écart-type de référence [10]. P/A Z-score <- 2 ET, T/A Z-score < - 2 ET et P/T Z-score < - 2 ET étaient considérés comme une insuffisance pondérale, un retard de croissance et une émaciation respectivement.

**Collecte et analyse des données:** Les données ont été collectées à partir de l'interview des parents lors de la consultation et le recours aux fiches de consultation. La saisie et l'analyse des données ont été faites à l'aide du logiciel SPSS 11.0 et Epi Info version 3.5.1 et Excel 2007. Pour la comparaison de proportion, le Chi carré corrigé de Fisher était utilisé avec un seuil de signification de p < 0,05.

## Résultats

**Caractéristiques cliniques et biologiques des enfants infectes par le VIH:** notre population était constituée de 83 enfants dont 45 garçons (54, 2%) et 38 filles (45,8%). L’âge moyen était de 34,1 mois avec des extrêmes de 6 et 59 mois. Presque la totalité des enfants étaient nés des mères VIH séropositives soit 95,7%. Par rapport à la découverte de l'infection, elle était motivationnelle dans 78,3% et 21,7% de façon fortuite. Le poids moyen était de 11,6±4,1 kg avec un minimum de 5 kg et un maximum de 22 kg. Le taux d'Hg moyen était d'environ 9,8±2,0 g/dl avec un minimum de 3,7 g/dl et un maximum de 16,2 g/dl. La prévalence globale de l'anémie était de 69,9% (n = 58) dont 36,1% avaient une anémie modérée et 2,4% une anémie sévère.

**Figure 1:**
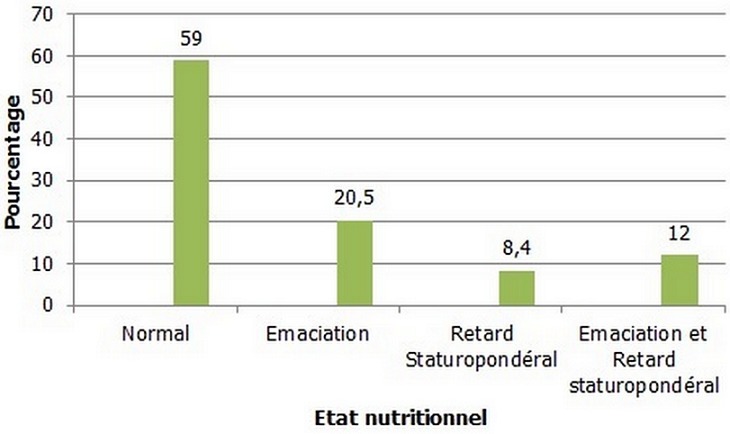
État nutritionnel en fonction du taux d'hémoglobine

**Prévalence de la malnutrition chez des enfants examinés:** la prévalence de la malnutrition globale était de 60,2% dont 8,4% des enfants présentaient une malnutrition sévère (Tableau 1). Par rapport à la catégorie de la malnutrition, 20,5% des enfants malnutris présentaient une émaciation (P/T Z-score <- 2), 8,4% un retard de croissance (T/A Z- score <- 2) et 12% présentaient à la fois une émaciation et un retard de croissance (Figure 1). La fréquence comparée de la malnutrition aigüe ((P/T < m - 2 ET) et chronique ((T/A < m - 2 ET) a montré que la malnutrition aigüe prédominait dans les tranches d’âge de 24-35 mois et 48-59 mois. La malnutrition chronique intéressait plus de la moitié des enfants au-delà de 12 mois (Tableau 2).

**Tableau 1:** État nutritionnel des enfants selon la classification de Gomez

Etat nutritionnel (P/A)	n	%
Bon état nutritionnel	33	39,8
Malnutrition au stade 1	26	31,3
Malnutrition au stade 2	17	20,5
Malnutrition au stade 3	7	8,4

Il ressort de ce tableau que 60,2% des enfants sont malnutris dont 8,4% de façon sévère (stade 3). L'insuffisance pondérale légère (malnutrition stade 1) à modérée (malnutrition stade 2) intéressait 51,8% des enfants

**Tableau 2:** La fréquence comparée des malnutritions aigue et chronique par tranche d’âge

Tranches d’âge (mois)	MPC aiguë (%) (P/T < m - 2 ET)	MPC chronique (%) (T/A < m - 2 ET)
6-11	0	50
12-23	13,3	60
24-35	23,5	63,2
36-47	8,3	50
48-59	23,1	66,7

Il ressort de ce tableau que la malnutrition aigüe prédominait dans les tranches d’âge 24-35 et 48-59. La malnutrition chronique intéressait plus de la moitié des enfants au-delà de 12 mois

**Catégories de la malnutrition en fonction du stade clinique et de l'anémie:** L'insuffisance pondérale (P/A Z-score <- 2) était associée de façon significative au stade avancée de l'infection à VIH (p = 0,002). De même, le retard de croissance (T/A Z-score <- 2) y était associé (p = < 0,007) ainsi que l’émaciation (p = 0,046) (Tableau 3). L'anémie (p = 0,6) n’était pas associée significativement au déficit nutritionnel (insuffisance pondérale, retard de croissance et émaciation) chez ces patients infectés par le VIH (Tableau 4).

**Tableau 3:** Étude de l’état nutritionnel en fonction du stade clinique de l'OMS

Indices anthropométriques		Stade clinique				P
		1	2	3	4	
		%	%	%	%	
P/T	<-2ET	26,7	26,7	40,0	6,7	0,046
	>-2ET	50,0	35,3	13,2	1,5	
T/A	<-2ET	28,9	42,2	24,4	4,4	0,007
	>-2ET	65,8	23,7	10,5	0	
P/A	<-2ET	25,0	32,1	39,3	3,6	0,002
	>-2ET	56,4	134,5	7,3	1,8	

Le tableau 2 montre que l'insuffisance pondérale (P/A), le retard de croissance (T/A) et l’émaciation (P/T) étaient associés de façon significative à la sévérité de l'infection VIH

**Tableau 4:** État nutritionnel en fonction du taux hémoglobine

Etat nutritionnel	Hg ≥ 11 g/dl n(%)	Hg 11g/dl n(%)	P
Normal	11(33,3)	22(66,7)	ns
Déficitaire	14(28,0)	36(72,0)	

L'analyse de ce tableau montre que l’état nutritionnel des enfants n’était pas associé à la survenue de l'anémie **(p = 0,6)**

## Discussion

Ce travail a évalué l’état nutritionnel des enfants âgés de 6 à 59 mois infectés par le VIH mais naïfs au traitement antirétroviral.

### Malnutrition globale

Dans notre travail, la prévalence globale de la malnutrition était de 60,2% dont 8,4% de malnutrition sévère. Au vu de ces résultats, la situation nutritionnelle se révèle précaire à Lubumbashi. La RDC connaît des taux élevés de malnutrition. En 2007, le focus humanitaire rapportait des taux allant jusqu’à 30% [11] et le programme national de lutte contre le VIH (PNLS) signalait une prévalence de 38,2% d'enfants congolais de moins de 5 ans souffrant d'un arrêt de croissance imputable à une carence nutritionnelle chronique et 16% d'enfants de moins de 5 ans souffrant d'une malnutrition aiguë sur un terrain d'infection à VIH [12]. Nos résultats confortent certains travaux dont celui de Mitangala Ndeba et al. (2008) qui ont trouvé 62,1% de malnutrition globale et 29,6% de malnutrition sévère chez les enfants non infectés par le VIH dans la région du Kivu (RD Congo) [13]. Dans le contexte d'infection à VIH au Malawi, Rogerson et al. [14] ont signalé en 2004 une prévalence plus faible de malnutrition que la nôtre soit 40%. Cette prévalence élevée de la malnutrition dans notre milieu s'expliquerait par le fait que la prise en charge nutritionnelle n'existe presque pas, la découverte de l'infection à VIH est souvent motivationnelle et tardive dans notre milieu soit 78,3% des cas et à un moment où il y a déjà une détérioration importante de l'immunité cellulaire chez ces enfants. Ces observations rejoignent celles de Njom Nlend (2007) qui signale une prévalence élevée de la malnutrition globale chez les enfants infectés par le VIH pouvant aller jusqu’à 90% et une prévalence élevée de l'infection à VIH chez les enfants malnutris [15]. Notre travail corrobore ces résultats en signalant une association positive entre la malnutrition globale et le stade avancé de l'infection à VIH. Les enfants nés de mère infectée par le VIH sont plus souvent prématurés et de faible poids de naissance. Une proportion élevée des enfants nés de mères VIH séropositives dans notre travail était de 95,7% et le poids moyen de ces enfants était de 11,6±4,1 kg dans notre travail. Ce qui peut aussi expliquer une prévalence élevée de la malnutrition. Plusieurs études antérieures avec divergentes opinions ont analysé la corrélation VIH maternel et croissance fœtale [16]. Les études menées dans les pays développés n'ont pas montré de différence entre les paramètres anthropométriques à la naissance des enfants de mères séropositives versus séronégatives. Par contre, dans les pays en voie de développement, il a été noté une réduction de ces paramètres chez les nouveau-nés de mères séropositives. Face à ces controverses, il s'avère nécessaire d'entreprendre une étude prospective permettant de bien évaluer l'impact de l'infection à VIH sur l’état nutritionnel.

### Emaciation

Chez l'enfant infecté par le VIH, l'amaigrissement représente une complication majeure au cours de l’évolution de la maladie. Dans notre travail, 20,5% des enfants présentaient une émaciation. Cette prévalence est similaire à celle de la RDC [17] soit 10,0%. Par contre, Mukalay et al. [18] ont signalé en 2009 à Lubumbashi chez les enfants séronégatifs au VIH âgés de moins de 5 ans une prévalence de l’émaciation de 3,8% inférieure à la nôtre. En somme, la prévalence de l’émaciation de notre étude parait élevée. Ceci pourrait être lié à une série des facteurs notamment le diagnostic tardif de l'infection à VIH et la prise en charge nutritionnelle déficiente. S'agissant de l'infection à VIH, notre travail a révélé une association positive entre l’émaciation et le stade avancé de l'infection à VIH. Il faut signaler que le contexte familial précaire favoriserait la malnutrition surtout que 39,8% de ses enfants sont orphelins de l'un de deux parents.

### Retard de croissance

En considérant l'indicateur taille pour âge, le retard de croissance concernait 8,4% des enfants de notre série. Cette prévalence est inférieure à celle de Kimani M et al. (2011) qui ont trouvé 18% en Afrique du sud [19]. Par contre Mukalay et al. [18] ont signalé à Lubumbashi chez les enfants séronégatifs au VIH âgés de moins de 5 ans une prévalence plus élevée que la nôtre soit 33,5%. De même, Beau et al. [20] au Cameroun (1997), Anita Shet et al. [21] en Inde (2009) et Arpadi [22] aux Etats-Unis (2000) ont trouvé respectivement 66%, 46% et 50%. Notre faible prévalence serait probablement liée à une petite taille de notre échantillon. De toute évidence, l'infection par le VIH affecte négativement l′issue de la grossesse et les nourrissons nés de femmes séropositives ont un poids et une taille inférieurs à la naissance. Les infections du tractus gastro-intestinal et la malabsorption des glucides, des lipides et des protéines sont communes dans l'infection à VIH, mais aucune relation entre ces troubles et une mauvaise croissance n'a été démontrée dans notre milieu. De même, la réplication du VIH empêcherait la croissance et sa suppression semblerait avoir un effet favorable sur la croissance pondérale [22]. Djadou et al. évaluant l'efficacité du TAR, ont signalé un taux de malnutrition aiguë sévère qui est passé à 56%, 47% et 25% respectivement à 3, à 6 et à 12 mois de traitement ARV avec un gain pondéral moyen de 860 g (inférieur à -3 ET), 1 550 g (compris entre -3ET et -2ET) et 1 270 g (compris entre -2ET et -1ET) respectivement à 3, à 6 et à 12 mois de traitement [23]. Des investigations approfondies sont nécessaires pour évaluer l’état nutritionnel sous traitement antirétroviral (ARV) dans notre milieu et leur interaction avec les parasitoses intestinales.

Dans les laboratoires bien équipés, la progression du VIH est essentiellement contrôlée par le nombre de CD4 (chez les enfants, en pourcentage des CD4) alors que dans les pays à ressources limitées comme le nôtre, la surveillance des CD4 n'est pas régulièrement assurée et souvent, les décisions de gestion sont guidées par le stade clinique de l'infection. Dans notre travail, le retard de croissance (p = 0,01), l'insuffisance pondérale (p = 0,002) et l’émaciation (p = 0,046) étaient associés à l’état avancé de l'infection à VIH. La malnutrition peut être une conséquence directe de l'infection à VIH, de la survenue des IO et des conditions de vie précaire. Anita Shet et al. ont signalé une association positive seulement avec l'insuffisance pondérale (p = 0,019) mais le retard de croissance (p = 0,06) et l’émaciation (p = 0,5) n’étaient pas du tout associés au stade avancé de l'infection à VIH [21]. En 2002, Dreyfuss et al. ont montré que chez des sujets infectés présentant une ou des carences nutritionnelles, il existait une dégradation des signes cliniques et une progression vers le stade SIDA [24]. Kimani et al. (2011) ont signalé une prévalence du retard de croissance plus élevée chez les enfants VIH positifs (29%) que les enfants séronégatifs pour le VIH (18%). La prévalence de l′insuffisance pondérale était de 10% (n = 67), tandis que celle de l’émaciation était de 7% (n = 44) [19].

Aucune association positive n'a été trouvée entre la survenue de l'anémie et les troubles de croissance dans notre travail. Par contre, Anita Shet et al ont rapporté que l′anémie est associée de façon significative à l′insuffisance pondérale et au retard de croissance, mais pas à l′émaciation [21]. Les troubles de croissance et une faible résistance aux infections sont aussi d'importantes conséquences de l'anémie, particulièrement celle due à la carence en fer. L'anémie contribue aussi très fortement à la morbidité et à la mortalité infanto-juvénile. De même, Soumaïla Mariko et al. (2009) ont rapporté au Mali des proportions d'enfants présentant des problèmes de retard de croissance et des problèmes de maigreur beaucoup plus élevées parmi ceux qui sont anémiques que parmi ceux qui ne l’étaient pas. De ce fait, ils ont constaté donc qu'il y avait une relation entre l'anémie et les troubles de croissance plus particulièrement le retard de croissance et l’émaciation [25]. Des études prospectives permettront d’évaluer l'interaction entre ces eux facteurs dans notre milieu. La nature transversale de l’étude ne nous a pas permis d’évaluer les carences nutritionnelles dans le contexte de l'infection à VIH en comparaison avec les enfants non infectés. Nous n'avons pas recherché les parasitoses intestinales mais le contexte géographique et la situation alimentaire précaire dans notre milieu contribuent en grande partie au développement des maladies infectieuses et parasitaires.

## Conclusion

Dans notre étude 60,2% ont présenté une malnutrition dont 8,4% au stade 3 de l'OMS. Le retard de croissance seul et associé à la malnutrition intéressaient 20,4%. Les enfants au stade avancé de l'infection à VIH en sont plus affectés. Ce travail conforte l'idée que l'infection par le VIH provoque et/ou aggrave les déficits nutritionnels de l'enfant. La prise en charge nutritionnelle s'impose donc chez les enfants malnutris et face à l'infection à VIH, il faudrait entamer la trithérapie le plus rapidement possible étant donné l'immunodépression sévère observée chez ces enfants.
